# Study of the Tooth Contact Pattern for Double-Enveloping Worm Gear

**DOI:** 10.3390/ma18173997

**Published:** 2025-08-26

**Authors:** Adam Kalina, Piotr Połowniak, Mariusz Sobolak

**Affiliations:** Faculty of Mechanical Engineering and Aeronautics, Rzeszów University of Technology, 35-959 Rzeszów, Poland; akalina@prz.edu.pl (A.K.); msobolak@prz.edu.pl (M.S.)

**Keywords:** double-enveloping worm gear, gear mesh, contact pattern, two-sheet type pressure film, computer image processing

## Abstract

This paper presents a comprehensive analysis of contact patterns in globoid worm gears with rectilinear axial tooth profiles. Two distinct methods are employed: a CAD-based simulation, and experimental techniques including two-sheet pressure measurement films. The CAD-based method for determining the contact pattern can be successfully applied to evaluate the form, size, and position of the contact pattern. Experimental results qualitatively confirm the theoretical predictions, although measured contact areas tend to be smaller due to incomplete filling and fabrication inaccuracies. The developed models offer reliable tools for evaluating tooth contact in worm gear design, potentially reducing prototyping costs. The proposed methods establish a solid foundation for further research and improvement of contact performance in double-enveloping worm gear systems.

## 1. Introduction

Gears are widely used across various industries due to their numerous advantages. The study of gear mechanisms is also of significant interest to research centres. A critical criterion for evaluating the proper operation of a gear transmission is its contact pattern. Analysing the contact pattern allows the assessment of meshing quality, optimisation of gear design, improvement of manufacturing accuracy, and ultimately enhancement of gear lifespan. Geometrically, the contact pattern is approximated as the vicinity of a theoretical contact point or line. This region is typically defined where the distance between mating surfaces falls below a specified threshold, often corresponding to the elastic deformation between contacting teeth. During actual gear operation, the real contact pattern slightly deviates from the ideal geometric one due to elastic deflections, machining errors, surface roughness, and assembly inaccuracies. Experimental studies, often performed under unloaded or lightly loaded conditions, reveal that the contact pattern can exhibit various shapes depending on gear type, manufacturing accuracy, assembly quality, and wear level [1]. Based on theoretical analyses and experimental tests, the location, shape, and size of the contact area are evaluated.

In both scientific research and engineering practice, three main groups of methods are used for analysing gear meshing behaviour:

1. Theoretical methods include analytical and numerical approaches. enabling the determination of contact points, contact lines, and their trajectories. The finite element method (FEM) is particularly valuable for analysing the influence of elastic deformations on contact location and corresponding stress distribution.
2. Simulation-based methods, primarily utilizing Computer-Aided Design (CAD) tools, involve analysing 3D models of gear sets for mutual intersection of tooth surfaces. CAD-based methods facilitate the visualization of the contact pattern throughout meshing and the definition of its geometric characteristics. Specialized Computer-Aided Engineering (CAE) tools (e.g., KISSsoft, KIMOS) further support qualitative evaluation of contact quality.
3. Experimental methods include techniques such as the widely used marking compound method, which provides a visual representation of the actual contact area.

Contact pattern analysis is a well-established subject of research for many types of gear drives. Numerous publications address investigations for cylindrical gears [2,3,4,5,6,7], bevel gears [8,9,10,11,12,13], and cylindrical worm gears [14,15]. Other specialized gear types, such as conical worm drives [16], hypoid gears [17], and face-milled hypoid gears [18], have also been investigated regarding their contact patterns. However, analyses of the contact pattern for globoid worm gears are lacking.

The double-enveloping hourglass worm drive has gained widespread application due to several advantages, such as multi-tooth engagement, which improves load distribution, and double-line contact, where the location of the contact lines provides more favourable lubrication conditions [19,20]. However, this type of worm gear requires high manufacturing precision and accurate alignment [21]. The double-enveloping worm gear can function either as a kinematic or a load-transmitting drive. It is widely utilized in aerospace, marine, and various industrial systems [22]. One of the most well-known variants is the Hindley worm gear, called also a TA worm drive, which features a straight tooth profile in the central plane. The surface of the worm is machined using a lathe equipped with a straight-edged cutting tool. The mating worm gear is manufactured using a hob of hourglass, geometrically similar to the worm [23,24]. For this type of gear, TCA analyses have been conducted without taking contact pattern analyses into account.

Early foundational research on double-enveloping worm gears by Litvin considered a simplified geometrical analysis of TA worm drives [20]. Simon laid the groundwork for understanding load distribution in these gears, meticulously considering factors such as tooth deflection and manufacturing errors [25,26]. More recently, Zhao and collaborators have significantly advanced the field, primarily focusing on TA worm drives and various modifications. Zhao and Zhang explored novel methods for curvature analysis and their application to TA worm drives, which are essential for understanding of contact geometry [27]. Building on this, Zhao presented further meshing analyses for TA worm drives [28], while also delving into the complexities of various modifications on contact geometry [29]. Additionally, Zhao and Zhang introduced a computing method for induced curvature parameters in TA worm gear pairs, enhancing analytical tools for contact analysis [30]. Huai and Zhao proposed variable height modification in TA worm drives to eliminate constant contact lines and validated its effectiveness through detailed meshing analysis [31].

Mohan and Shunmugam investigated the geometrical aspects of double-enveloping worm drives (ZN type). They employed geometrical simulation of tooth generation of the worm wheel and proposed a simplified manufacturing method [32]. Similarly, Chen and Tsay developed mathematical models for this type of worm gear and analysed how gear parameters influence boundary and contact lines [33].

Other publications have focused on the development of new types of globoid worm gears, such as planar, double-roller, toroidal involute worm drives, and dual-torus hourglass worm drives [34,35,36,37,38,39,40,41,42,43,44], as well as on their manufacturing methods [45,46,47,48,49].

This study focuses on selected investigations and analyses of the contact pattern for a globoid worm gear with a straight tooth profile in the axial section (Hindley worm gear). A multifaceted approach is presented, combining CAD-based simulation and direct experimental techniques, including two-sheet pressure measurement films. The experimental method specifically utilizes computer processing of images obtained by scanning the sheets of pressure-measuring films manufactured by Sensor Products Inc. (Sensor Products Inc., Madison, NJ, USA) and FujiFilm (FujiFilm Europe GmbH, Ratingen, Germany). The experiment was conducted for a loaded gearbox. Furthermore, the size and position of the contact pattern were checked for all worm wheel teeth in mesh. Analysis of the results obtained aimed to demonstrate a convergence between the theoretical study and the experiment, using the area and width of the contact pattern as key criteria.

## 2. Materials and Methods

### 2.1. Kinematic System of Gear Mesh

**Figure 1:**
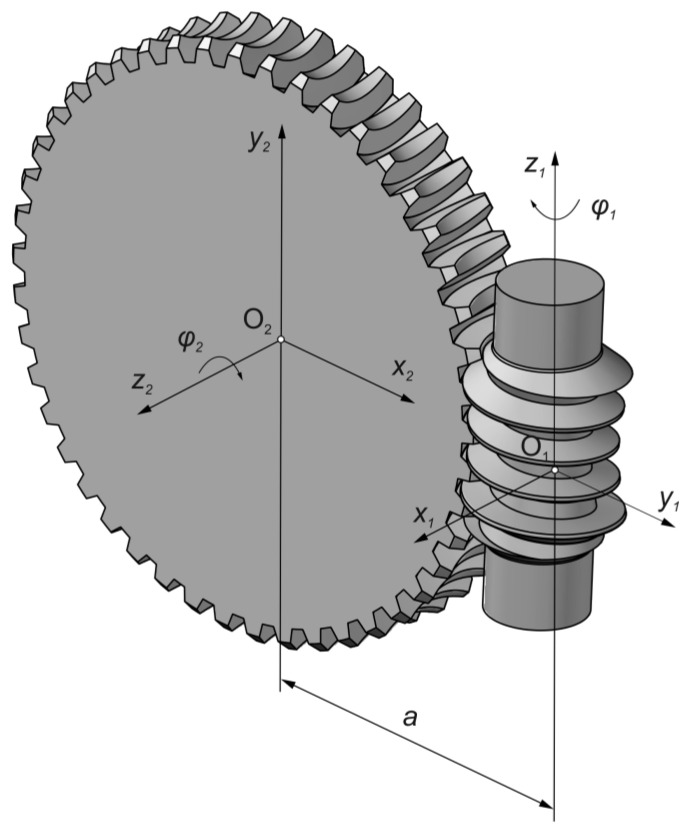
Kinematic system of a globoid worm gear.

The analysis concerns a double-enveloping worm gear with a straight axial tooth profile, commonly referred to as a Hindley worm gear. In this gear type, the worm surface is generated by a turning process using a lathe tool with a straight-edged blade. Figure 1 illustrates the kinematic configuration of the system.

The mechanism constitutes a gear pair with axes oriented at 90°, separated by a centre distance denoted as a. To describe the kinematic relationships within the gear pair, two coordinate systems were introduced: the first, ${x_{1},y}_{1},z_{1}$, was associated with the worm, and the second, ${x_{2},y}_{2},z_{2}$, was associated with the worm wheel. The origins of these coordinate systems are located at points $O_{1}$ and $O_{2}$, respectively. Parameter $\varphi_{1}$ for worm and $\varphi_{2}$ for worm wheel are applied to describe relative rotations during gear operation. The kinematic relationship between the angular parameters is defined by Equation (1), which expresses the transmission ratio.

(1) $$i=\frac{\varphi_{2}}{\varphi_{1}}$$

In the case of a left-hand threaded worm rotating about the $z_{1}$ axis by an angle $\varphi_{1}$ in the direction opposite to the trigonometric (counterclockwise) rotation, the worm wheel also correspondingly rotates about the $z_{2}$ axis by an angle $\varphi_{2}$ in the opposite trigonometric direction.

#### 2.1.1. Parameters of the Gear Set

**Figure 2:**
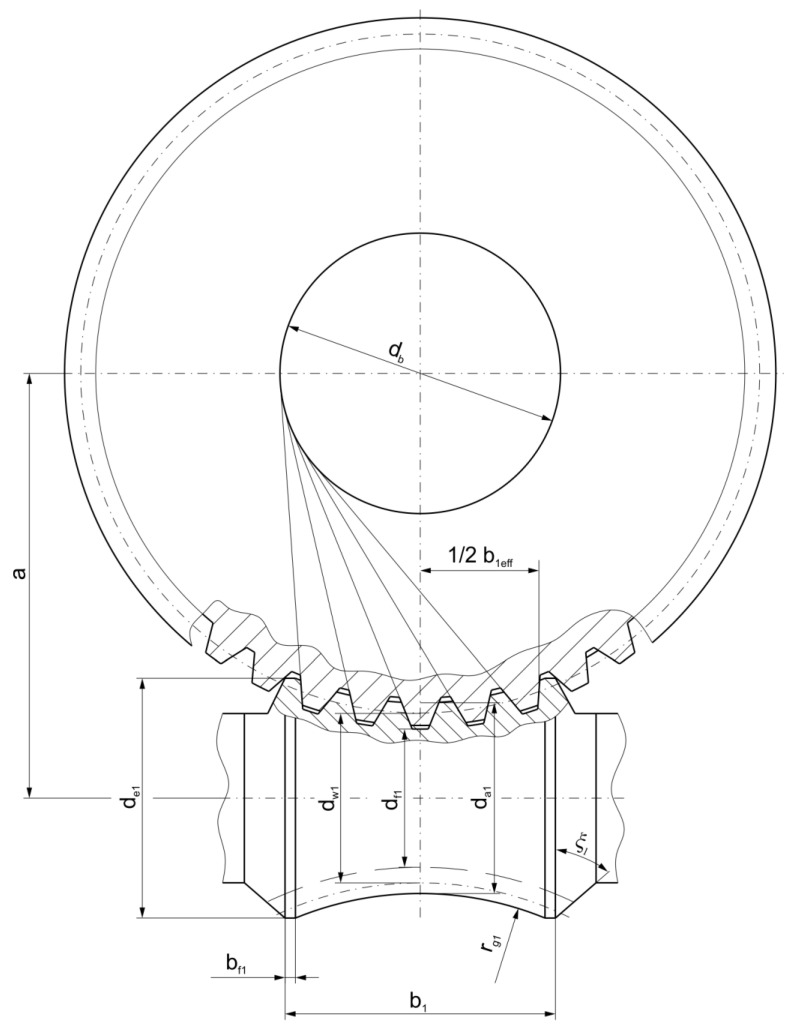
Gear pair shown in central plane.

**Figure 3:**
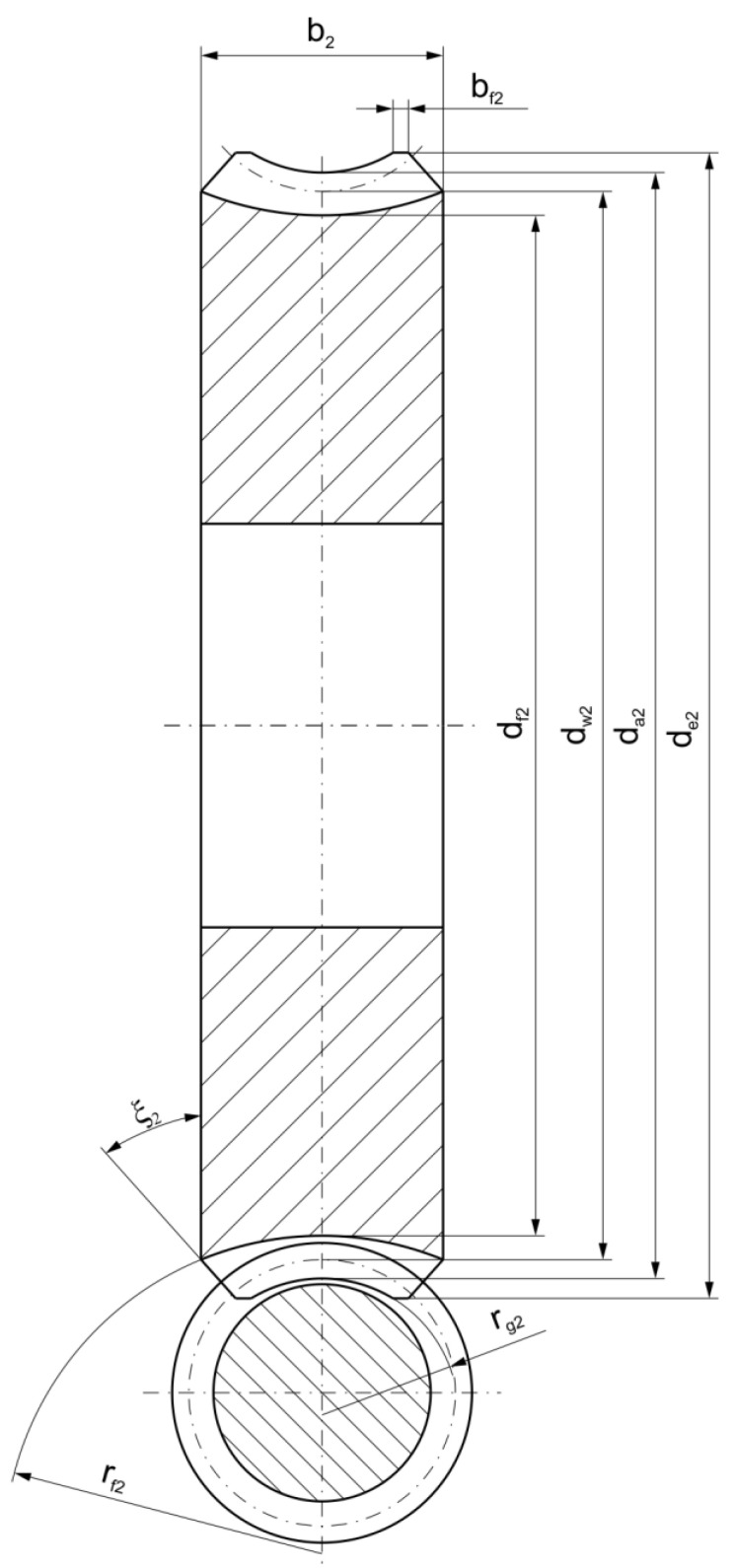
Gear pair shown in transverse plane.

Figure 2 and Figure 3 present the geometric parameters of the gearing, as detailed in Table 1.

For this type of gear design, proportions of the gear geometry are specified in the AGMA standard [50]. A distinguishing characteristic of double-enveloping worm gears with a Hindley worm is that their tooth profile in the axial cross-section is tangent to the base circle, denoted by the parameter $d_{b}$ (Figure 2). The analysis of the contact pattern for the double-enveloping worm gear is based on the parameters listed in Table 1.

#### 2.1.2. Contact Pattern Determined Using CAD-Based Method

**Figure 4:**
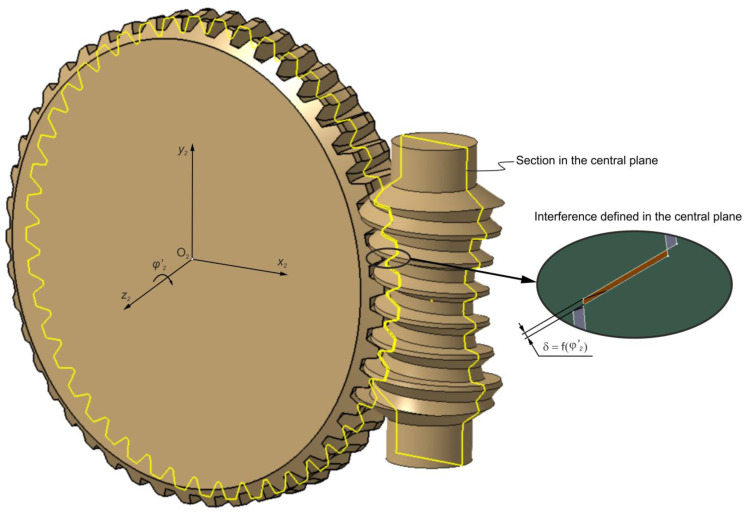
Interference of the worm gear models.

The tooth contact pattern can be developed within a CAD environment. The hourglass worm and worm wheel are modelled and assembled into a transmission gear, as illustrated in Figure 1. In the ideal gearbox model, manufacturing and assembly errors, as well as elastic deformations of the shafts and bearings, are not taken into account. Parameterization of the relative rotatory motion of the worm gear pair was introduced to enable tooth contact analysis over the entire cycle of worm rotation. The worm wheel model is rotated around the $z_{2}$ axis (Figure 1) by a specified angle ${\varphi^{\prime }}_{2}$, allowing its controlled interference with the worm thread. The magnitude of the interference is defined in the central plane by a value δ (Figure 4), which may correspond to the thickness of a marking compound layer.

During the simulation of the gear meshing, both components are incrementally rotated in discrete steps according to the gear ratio. The intersection between the worm and the worm wheel at a given position is determined, resulting in the generation of flake elements. The area of the contact pattern is calculated based on the surface area of these flake elements [1]. This method enables the analysis of the contact pattern for the entire cycle of worm rotation. Based on this method, it is possible to evaluate the shape, position and size of the contact pattern. Additionally, a graphical characterisation of the contact area size depending on worm position can be performed. An advantage of the CAD-based method is that the CAD model of the hourglass worm includes the full length of the thread, including the thread exit modification, while the worm wheel model reflects the geometry of the gear blank contour.

### 2.2. Experimental Procedure

In order to perform experimental studies of the contact pattern in the double-enveloping worm gear, a dedicated test stand was designed and physical gear pair models were manufactured. In a real gear system, the worm and worm wheel are typically made of steel and bronze, respectively. For experiment, the polymer materials were used, which are a common choice for prototype models because they significantly reduce manufacturing costs and production time.

#### 2.2.1. Globoid Worm Gear Manufacturing

**Figure 5:**
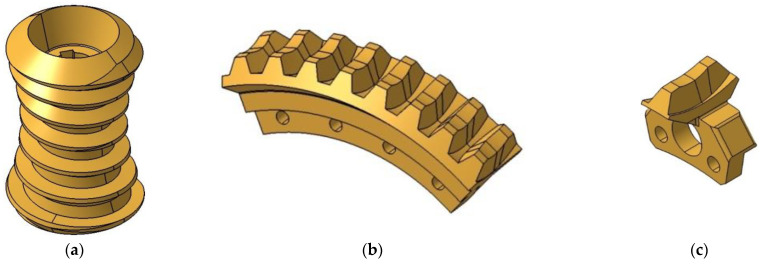
Prepared CAD models for rapid prototyping: (**a**) hourglass worm model; (**b**) worm wheel model with eight teeth; (**c**) single-tooth worm wheel model.

CAD solid models of the double-enveloping worm gear were created in a CAD environment CATIA V5R21 (Dassault Systèmes, Vélizy-Villacoublay, France) based on the methodology described in [51], and prepared for assembly on the test stand. These included a model of the hourglass worm (Figure 5a), a worm wheel model with eight teeth (Figure 5b), and a single-tooth worm wheel model (Figure 5c).

The CAD models were used to fabricate components from transparent photopolymer resin using the PolyJet 3D printing method, classified as a rapid prototyping (RP) technique. RGD720 material was used for the printed models, and RGD705 for the support structures. The material parameters are presented in Table 2.

**Figure 6:**
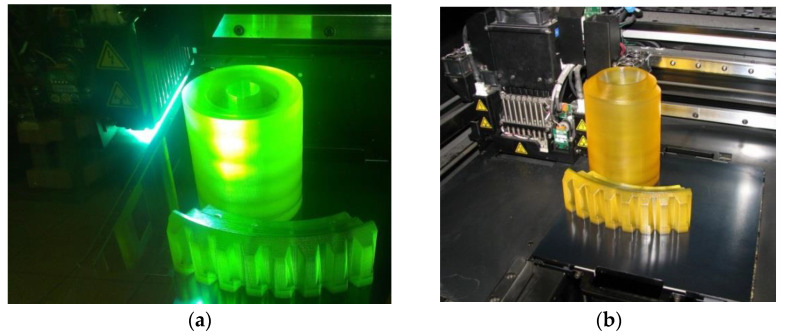
Models printed with support structures on the Objet Eden 260 V: (**a**) during printing; (**b**) after printing.

**Figure 7:**
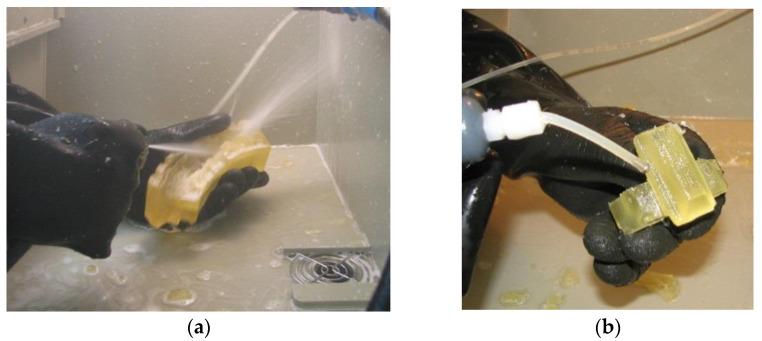
Prototype gearbox components cleaned in a high-pressure washer: (**a**) worm wheel with eight teeth; (**b**) single-tooth worm wheel.

After printing (Figure 6) on an Objet Eden 260V 3D printer, the models were cleaned in a high-pressure washer (Figure 7).

#### 2.2.2. Test Stand

**Figure 8:**
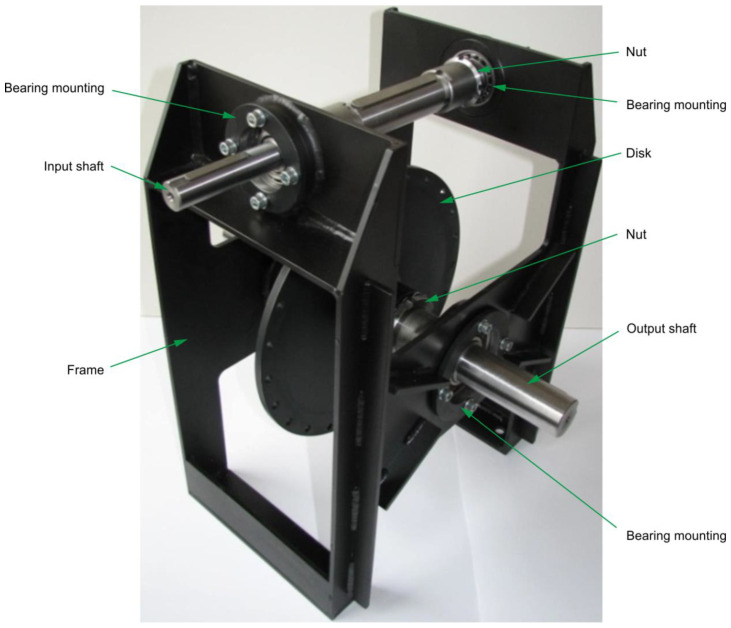
Test stand for double-enveloping worm gear.

To verify the contact pattern results obtained from theoretical analyses, a physical test stand was designed and manufactured (Figure 8). The gear models (Table 1) were fabricated at a 1:1 scale and assembled on the test stand. The worm prototype was mounted on the input shaft, with axial alignment enabled using bearing nuts. The worm wheel prototype was fixed to a disc mounted on the output shaft with axial adjustment capability. Torque input and output could be applied from both ends of the shafts.

**Figure 9:**
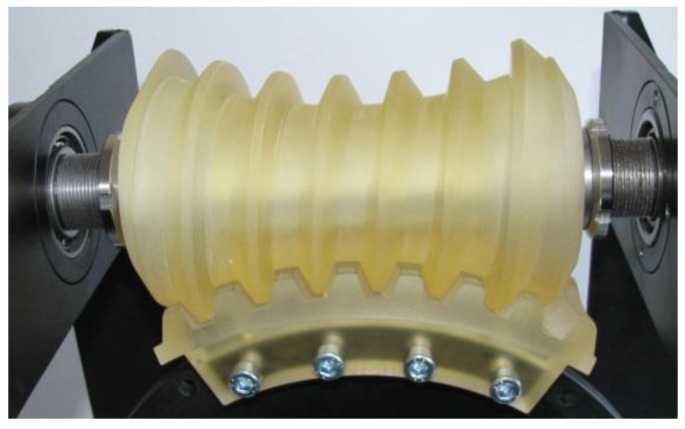
Test stand with assembled prototype models of the double-enveloping worm gear.

The prototypes of the double-enveloping worm gear were assembled on the test stand (Figure 9).

#### 2.2.3. Experimental Method: Chalk Paint, Liquid Tracing and Deformation Freezing

One experimental approach involves the use of chalk paint as a marking compound applied to the worm wheel teeth, and then the gear is meshed under a small load. As a result of the contact, portions of the paint are abraded, revealing the contact zone. Depending on the type of motion, this method can yield either single instantaneous contact patterns (resulting from small, reversible angular displacements of the worm) or the total contact pattern (produced by multiple passes of the worm wheel teeth across the worm thread).

Other experimental methods may be applicable to different types of gears. One such method is direct observation of the contact pattern between meshing teeth. This is possible when gear models are fabricated from transparent materials [53]. In this approach, a liquid such as oil or water is used as the marking medium. The resulting liquid meniscus corresponds to a contour line of equal distance between the meshing surfaces and provides a visual representation of the instantaneous contact pattern [4]. However, due to the complex structure of the gearbox and limited visibility, this method is not suitable for double-enveloping worm gears.

Another method is deformation freezing [54], which involves placing a deformable material, such as an acrylic layer, into the gear mesh and applying a load. Once the material has set, the gears are disengaged, and the momentary contact pattern can be analysed. As with the previous method, deformation freezing is not applicable to globoid worm gears.

#### 2.2.4. Method Based on Pressure Measurement Using Two-Sheet Type Film

Pressure-measuring films are specialized sheets designed to quantify pressure distribution. They are available in two variants: a two-sheet type (e.g., Sensor Products Inc. SPF-A) and a mono-sheet type (e.g., FujiFilm Prescale Super High Pressure-HHS). Each type is further divided into subtypes optimized for different pressure ranges. Two-sheet films are capable of measuring lower pressures (from 0.006 to 50 MPa), whereas mono-sheet films are suitable for higher pressures, ranging from 10 to 300 MPa [55,56].

These films are widely used, especially in medical applications [57,58]. Although their primary function is pressure measurement, they are also used to determine contact areas, as demonstrated in previous studies [59,60]. Considering the results of the aforementioned research and the advantages of pressure-measuring films, such as their minimal thickness compared to strain gauges and their adaptability to various geometries by simple cutting, it was decided to use these films for measuring the contact pattern area in worm gear meshing.

**Figure 10:**
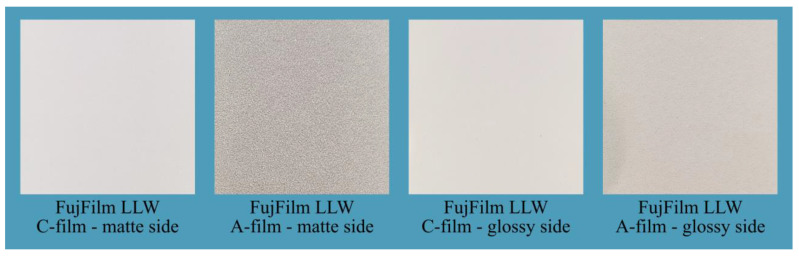
Prescale pressure measuring film manufactured by FujiFilm.

The presented method for determining the contact pattern is based on the use of two-sheet pressure-measuring films (Prescale pressure-measuring film) manufactured by FujiFilm. In this study, Super Low Pressure (LLW) films were used, which enable pressure measurements in the range of 0.5 to 2.5 MPa [55]. The system consists of two sheets: the A-film and the C-film, as shown in Figure 10.

**Figure 11:**
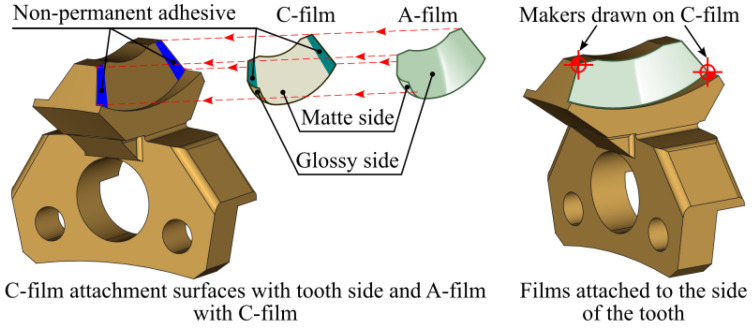
Method of attaching films to the side of the worm wheel tooth.

Prior to testing, both sheets were cut to the desired shape. The films were then placed on the mating surfaces such that their matte sides face each other. Due to the complex geometry of the globoid worm gear and the limited access to the meshing areas, the films were attached to the flanks of the worm wheel teeth, as shown in Figure 11.

As shown in Figure 11, the method requires the use of adhesive to temporarily bond the films to the worm wheel tooth. Adhesive was first applied to the matte side of the C-film and then bonded to the A-film. Next, the adhesive was applied to the worm wheel tooth flank, and the previously joined films were bonded using the glossy side of the C-film. Glue was applied in non-contact areas of the tooth to minimize its impact on the accuracy of the contact pattern measurement. Additionally, to simplify further analysis, markers were applied to the C-film at the locations indicated in Figure 11. These markers help identify the exact position of the contact pattern during scanning and image processing.

**Figure 12:**
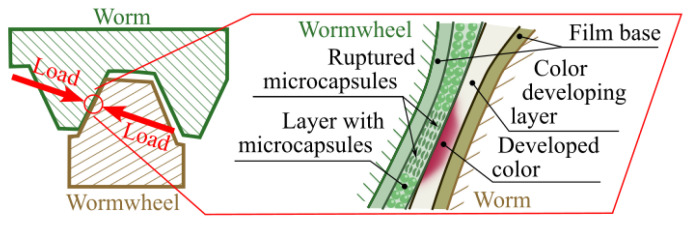
Rupture of the microcapsules caused by pressure during meshing.

The A-film has a layer of microcapsules containing an active substance. During meshing, the pressure causes the microcapsules to rupture, releasing this substance (Figure 12). The C-film, on its matte side, contains a colour-developing layer that reacts with the substance from the A-film. This reaction results in discoloration, and the intensity of the colour correlates with the pressure level at the contact area.

**Figure 13:**
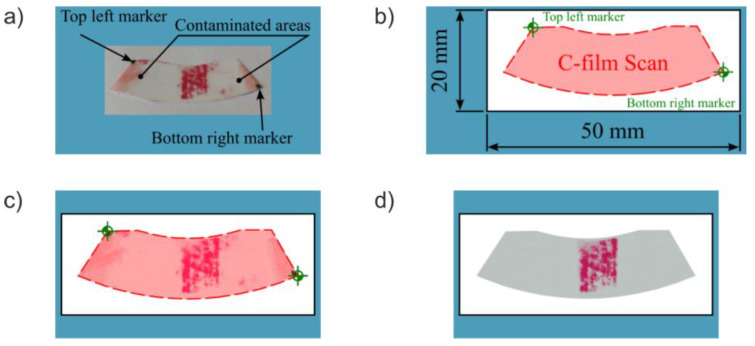
Sample preparation: (**a**) picture of C–film; (**b**) scan cropping template; (**c**) scanned and cropped C–film image; (**d**) final processed sample cleaned of glue residue, discoloration, noise, and markers.

According to the manufacturer, the recommended load time is at least 5 s for momentary loads and 120 s for continuous loads. Additionally, ambient temperature and humidity should be recorded during testing, as these factors influence the colour intensity of the C-film [55]. To enhance colour development, the developer sheet should be left exposed to ambient conditions for a specific time before scanning. Figure 13a shows an example of a developed C-film with a visible contact pattern and positioning markers.

The C-film is then scanned and cropped using a graphics program and the developed template (Figure 13b). This template includes a semi-transparent overlay with markers (corresponding to those in Figure 11 and Figure 13a) that allows precise alignment and cropping of the scan. The template is as small as possible to reduce image file size. All the coloured elements visible in the template, as well as the black frame, will be excluded from the final sample image. Figure 13c shows the aligned and cropped scan, Some elements of the template have been intentionally retained to illustrate the matching process. Remnants of contamination from the bonding process are still visible. Therefore, image pre-processing is applied to remove contamination and markers before analysis. The final cleaned image is shown in Figure 13d.

**Figure 14:**
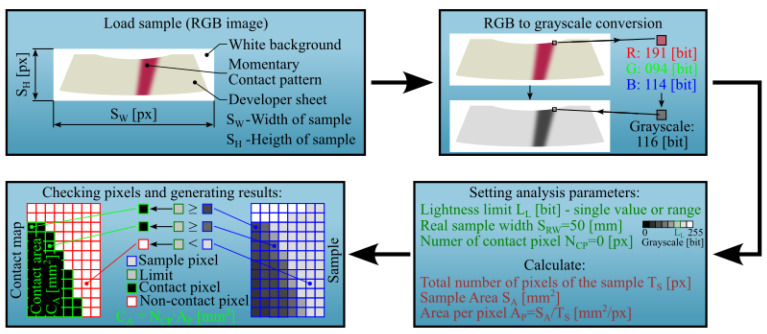
Simplified algorithm of sample image processing.

A dedicated software tool was developed to analyse the contact pattern. The program was written in MATLAB and is compatible with the MATLAB R2023b version (MathWorks, Inc., Natick, MA, USA). Its primary function is to generate a contact map and calculate the contact area. A simplified algorithm is shown in Figure 14.

After placing the images in the working directory and launching the program, the image dimensions ($S_{H}$ for height, $S_{W}$ for width in pixels) are read. The RGB image is then converted to greyscale. The actual width of the sample ($S_{RW}$ in mm) can either be entered manually or extracted from the filename. This width corresponds to the template shown in Figure 13b. The most important user-defined parameter is the lightness limit $L_{L}$, expressed in bits, which defines the grayscale threshold above which pixels are considered non-contact. It ranges from 0 (black) to 255 (white), although typical values are lower. A single value or range, e.g., [0, 150], can be specified. The number of total pixels in the image is calculated as:

(2) $$T_{S}=S_{H}\cdot S_{W}$$

With the known resolution and actual sample width $S_{RW}$, the total sample area $S_{A}$ in mm^2^ is calculated as:

(3) $$S_{A}=\frac{S_{RW}^{2}\cdot S_{H}}{S_{W}}$$

The area per pixel ($A_{P}$ in $\frac{mm^{2}}{px}$) is given by:

(4) $$A_{P}=\frac{S_{A}}{T_{S}}$$

After completing the calculations, the image analysis begins by comparing the brightness of individual pixels to a user-defined lightness limit $L_{L}$. If a pixel’s brightness is less than or equal to $L_{L}$, the parameter $N_{CP}$—representing the number of pixels classified as being in contact—is incremented by 1. This parameter is initialized to 0 at the start of each analysis. Simultaneously, a monochromatic image, referred to as the contact map, is generated. In this map, each black pixel corresponds to a pixel in the original image whose brightness is less than or equal to $L_{L}$. Upon completion, the program saves the contact map as a separate image and computes the contact area, expressed in $mm^{2}$, as follows:

(5) $$C_{A}=N_{CP}\cdot A_{P}$$

**Figure 15:**
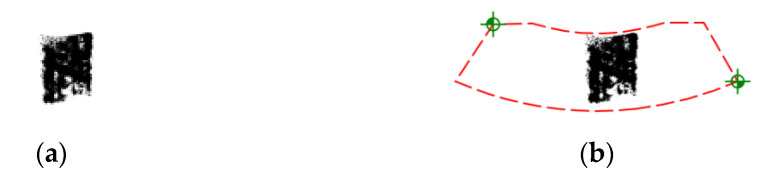
(**a**) Generated contact map; (**b**) Contact map with post-processed overlay frame corresponding to the template shown in Figure 13b.

Figure 15a presents a sample contact map, while Figure 15b shows the same map with a post-processed overlay frame used to identify the contact pattern on the worm wheel tooth flank. This frame corresponds to the template shown in Figure 13b. The advantage of the adopted solution, a monochromatic contact map overlaid with a marker frame, is its ability to combine images obtained from different worm wheel teeth and various worm positions. This allows for the determination of the total contact pattern.

**Figure 16:**
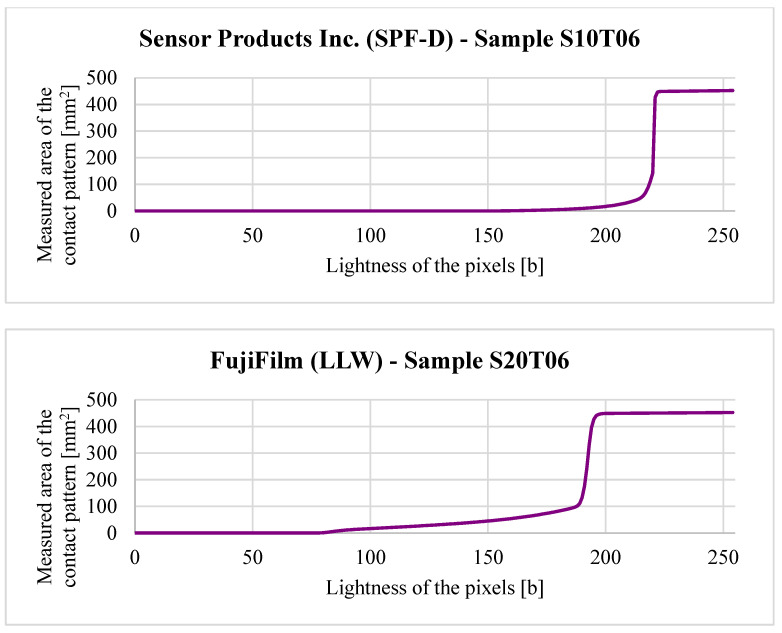
Effect of the lightness limit $L_{L}$ on measured contact area $C_{A}$.

If the user specifies a range for $L_{L}$, the program iterates through the values, generating corresponding contact maps and plotting the effect of $L_{L}$ on measured contact area (Figure 16).

**Figure 17:**
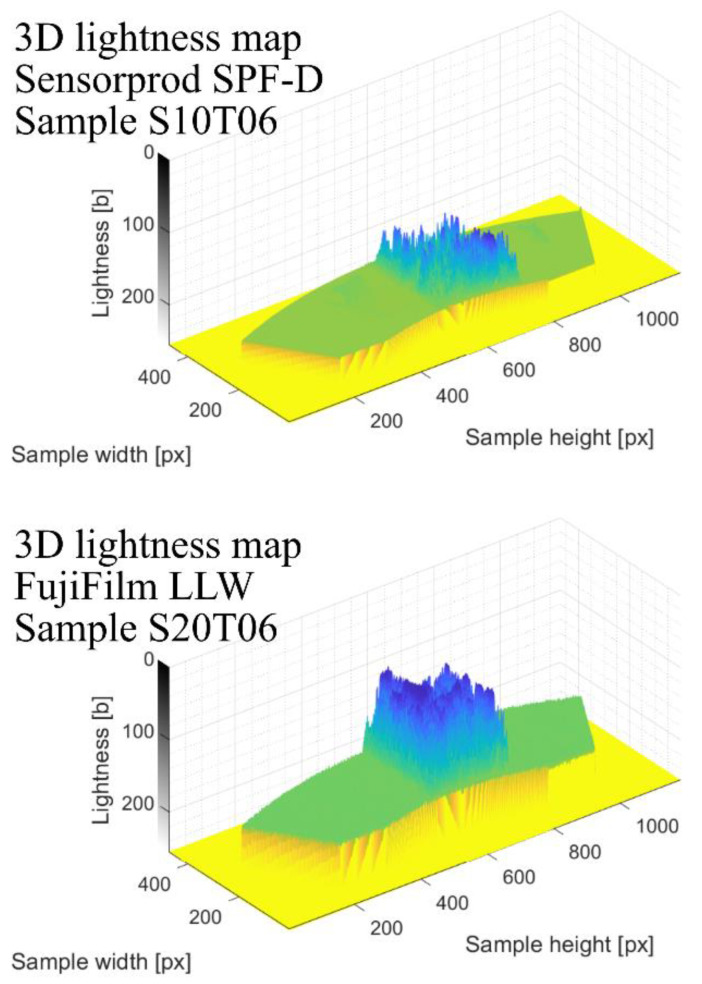
3D lightness maps of the scanned sample.

Finally, the program generates a 3D lightness map (Figure 17), where the vertical axis represents the brightness intensity of individual pixels.

This map assists users in selecting a suitable $L_{L}$ threshold, which depends on the film type, scanner settings, environmental conditions, and image pre-processing steps.

**Figure 18:**
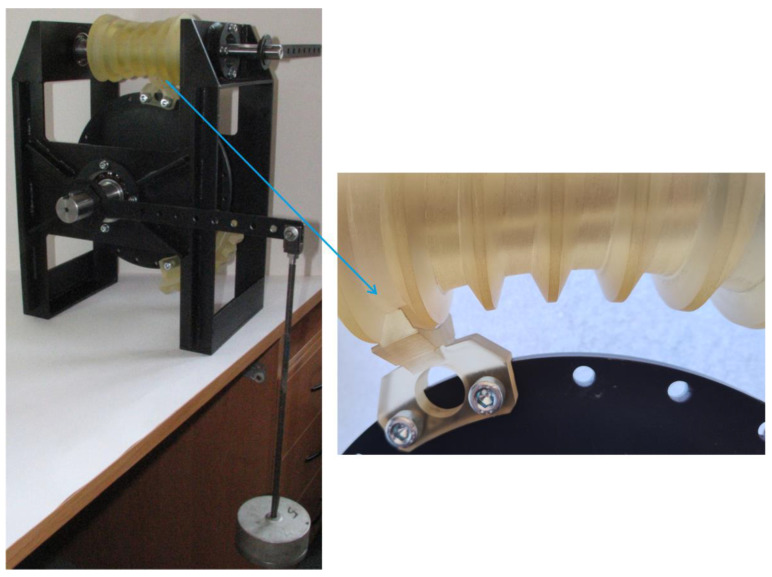
Test stand with assembled prototypes and pressure-measuring films installed under load.

Experimental testing using the two-sheet pressure film was conducted on a dedicated test rig (Figure 18). The worm was fixed and a torque of 36.2 Nm was applied to the worm wheel. This torque was primarily generated by weights suspended from a lever (of known length) attached to the worm wheel shaft. The torque resulting from the mass of the lever itself was also taken into account. Based on the manufacturer’s guidelines, the contact duration was set to 120 s. Ambient temperature and humidity were recorded at 22 °C and 58%, respectively. Prior to scanning, the films were conditioned in the same environment for 30 min. Scanning was performed using an Epson Perfection V600 Photo scanner (Seiko Epson Corporation, Owa, Suwa-shi Nagano-ken, Japan) at 300 dpi resolution.

## 3. Results and Discussion

This subsection presents the results of the contact pattern analysis for the double-enveloping worm gear, based on the parameters listed in Table 1.

### 3.1. CAD Analysis of Tooth Contact

**Figure 19:**
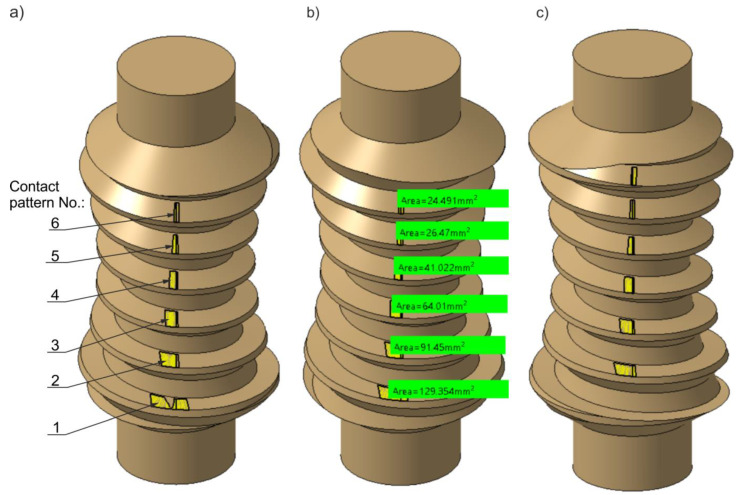
Instantaneous contact patterns shown on the worm tooth flank surfaces. Worm position: (**a**) $\varphi_{1}=36^{{}^{\circ}}$; (**b**) $\varphi_{1}=108^{{}^{\circ}}$ (with measured contact pattern areas); (**c**) $\varphi_{1}=288^{{}^{\circ}}$.

**Figure 20:**
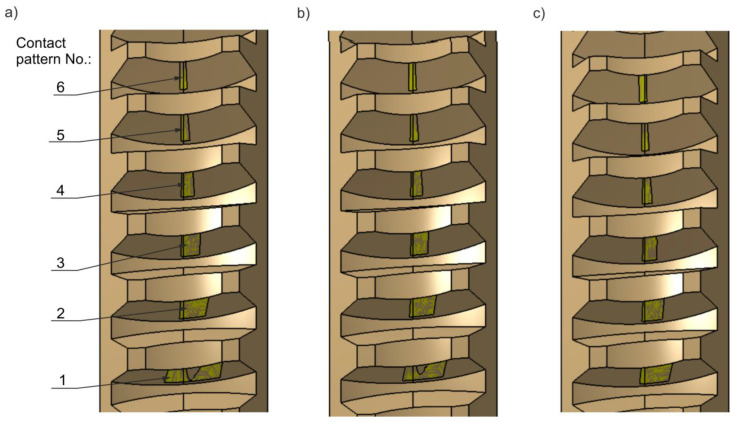
Instantaneous contact patterns shown on worm wheel tooth flank surfaces. Worm position: (**a**) $\varphi_{1}=36^{{}^{\circ}};$ (**b**) $\varphi_{1}=108^{{}^{\circ}}$; (**c**) $\varphi_{1}=288^{{}^{\circ}}$.

**Figure 21:**
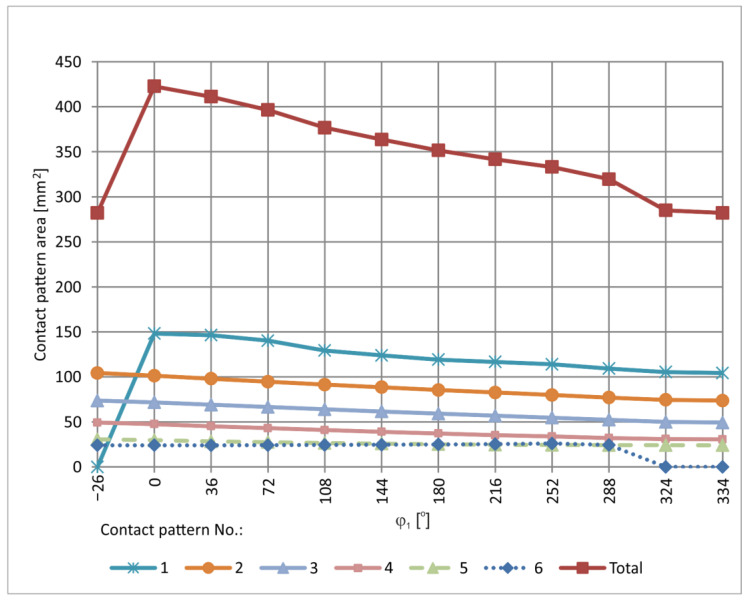
Variation of contact pattern area determined in the CAD environment.

The contact pattern investigated in the CAD environment was analysed for the value δ = 0.02 mm. It was determined for defined positions of the worm gear pair throughout the full cycle of worm rotation. Representative graphical results of the instantaneous set of contact patterns are presented in Figure 19 and Figure 20. These patterns are sequentially numbered, where No. 1 corresponds to the contact pattern at the beginning of the worm thread, and the final number corresponds to the last contact, near the end of the thread. In addition, a graphical representation of the contact area size as a function of worm rotation angle is provided in Figure 21. The diagram allows single contact patterns to be analysed or the cumulative contact pattern to be obtained by summing them. A quantitative analysis of how the contact area changes with worm position is presented in Table 3 and visualized in Figure 21. Table 3 lists the total area of the instantaneous contact patterns, denoted as Σ [mm^2^]. All values are rounded to one decimal place.

### 3.2. Experimental Method Using Chalk Paint

**Figure 22:**
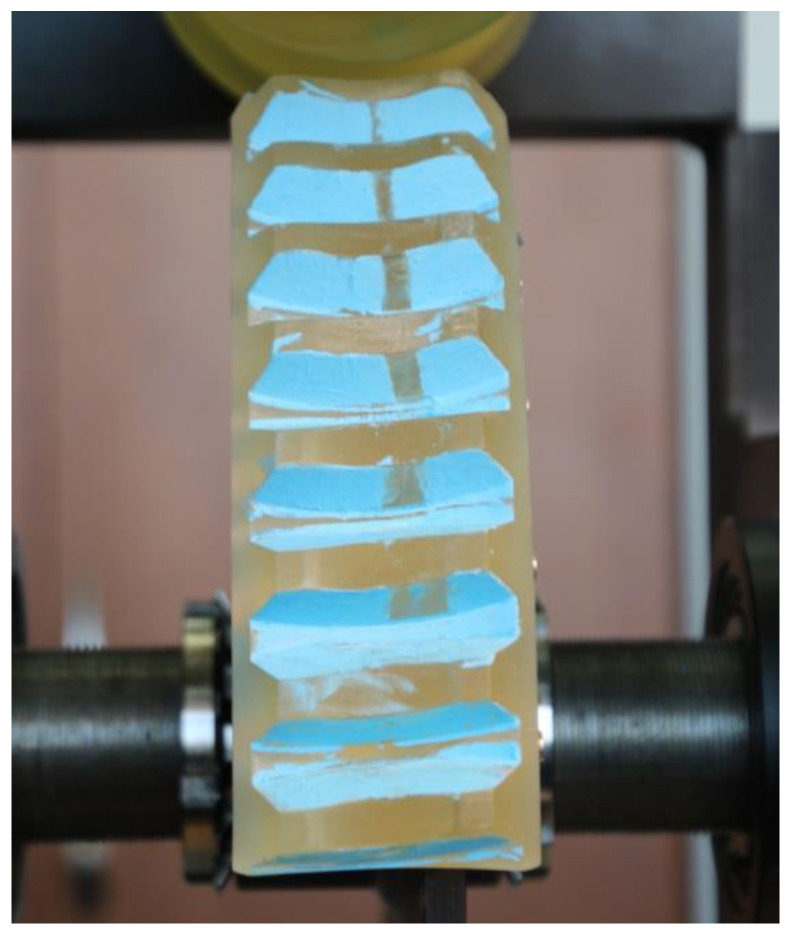
Instantaneous contact patterns obtained using the chalk paint method.

Based on the chalk paint experimental method, the shape and location of the contact pattern can be visually observed. Figure 22 presents a set of instantaneous contact patterns obtained during testing.

While determining the size of the contact pattern is problematic with this method, it proves useful for examining the shape and position of instantaneous contact regions on the tooth surfaces.

### 3.3. Analysis of Tooth Contact Pattern Using Two-Sheet Pressure Measurement Film

**Figure 23:**
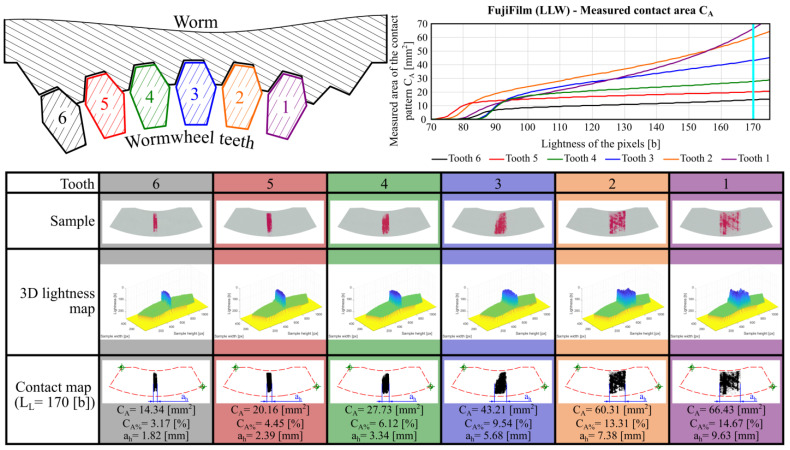
Measured contact pattern areas obtained using FujiFilm LLW pressure measurement films.

Figure 23 shows the results of the contact pattern analysis using FujiFilm LLW pressure measurement films for selected worm wheel teeth that were engaged in the meshing process.

**Figure 24:**
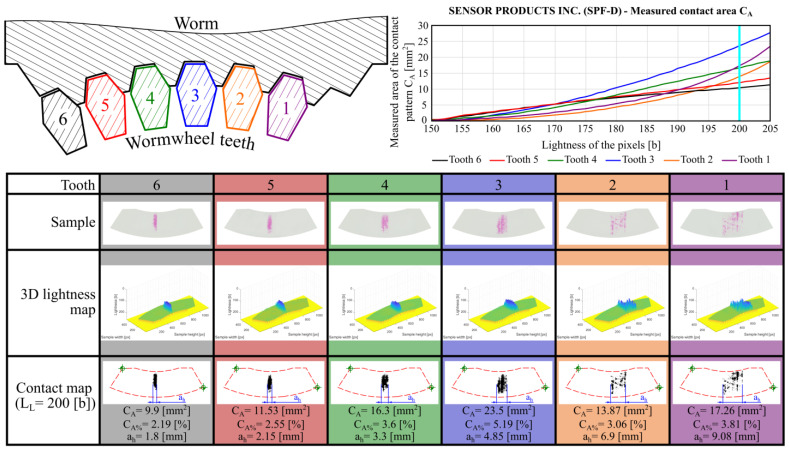
Measured contact pattern areas obtained using Sensor Products Inc. SPF-D films.

Additionally, for comparative purposes, control tests were conducted using pressure-measuring film manufactured by Sensor Products Inc. (Figure 24). The SPF-D film was used, which is designed for measuring higher pressure values (2.413–9.652 MPa) than LLW films.

The contact area fill rate is higher for LLW films than for SPF-D films, as LLW sheets are designed to measure lower pressure values.

The analysis of the obtained results indicates that the measured contact pattern areas are smaller than those determined using the previously described methods. This is because the method based on pressure measurement films is experimental in nature. Additionally, it is strongly influenced by the geometrical structure of the contacting surfaces, their shape, and the applied load. As a result, the obtained contact maps show outer contours of the contact patterns that are similar in shape and extent to those predicted by theoretical approaches. However, the internal contact regions are not completely filled. Furthermore, the contact pattern widths $a_{h}$ were also measured and are included in Figure 23 and Figure 24.

**Figure 25:**
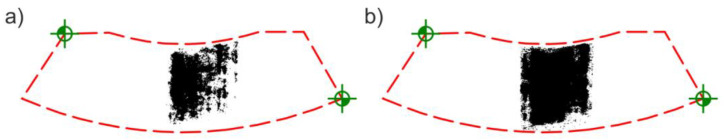
Total contact pattern, with: (**a**) Sensor Products Inc. SPF–D; (**b**) FujiFilm LLW.

Due to the monochromatic nature of the contact maps, it was possible to derive the overall contact pattern by summing the images acquired from each individual tooth. This determined total contact pattern is shown in Figure 25.

### 3.4. Comparison

The comparison of contact patterns obtained by CAD-based methods and by experimental methods using chalk paint and two-sheet pressure measurement film shows that the shape and location are similar.

In the CAD method, the flake body reflects the shape of the flanks of the worm and worm wheel. The CAD method takes into account the interference depth of the solids in the central plane. Generally, the theoretical results can be controlled by adjusting the parameters δ.

The experimental method using chalk paint is most suited to investigating the general shape and location of the contact pattern. Qualitative assessment shows that the shapes and locations of the instantaneous contact patterns correspond well with those obtained from CAD methods.

**Figure 26:**
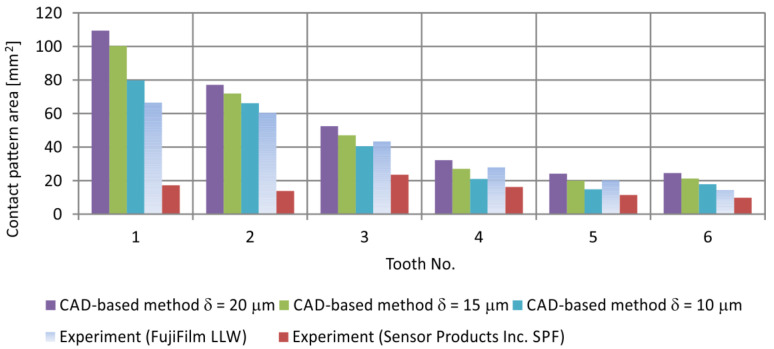
Comparison of the set of instantaneous contact pattern areas determined theoretically and experimentally for the gear set position $\varphi_{1}$ = 288°.

**Figure 27:**
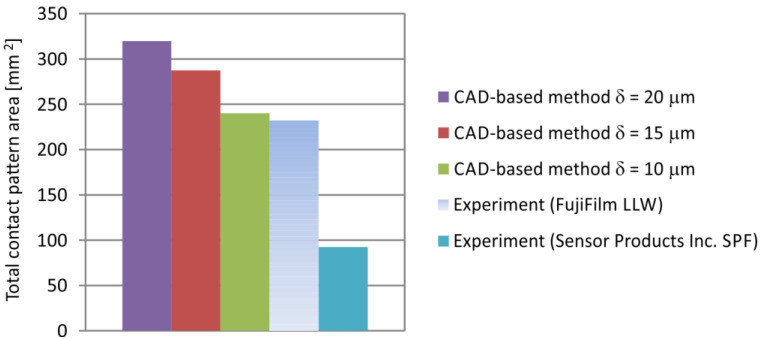
Comparison of the total contact pattern area determined theoretically and experimentally for the gear set position $\varphi_{1}$ = 288°.

The comparison of the contact pattern areas obtained by theoretical method and experimentally, using the two-sheet pressure measurement film for the investigated worm gear position ($\varphi_{1}=288^{{}^{\circ}}$), is presented in Figure 26 and Figure 27. The differences between experimental and CAD-based methods are mainly due to the incomplete filling of the contact regions in the experimental results, as well as inaccuracies resulting from surfaces fabricated by rapid prototyping and assembly errors. They also depend on the assumed parameter δ.

**Figure 28:**
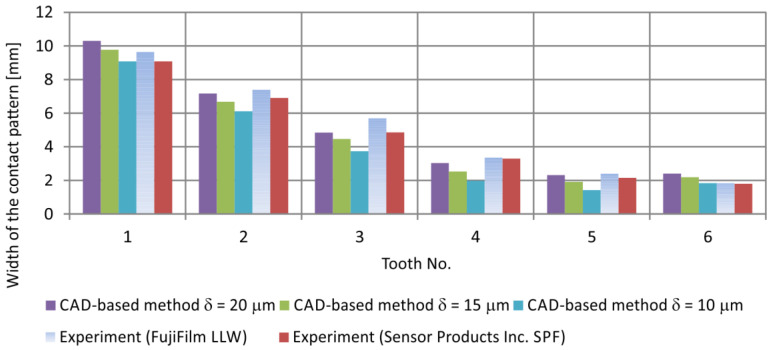
Comparison of contact pattern widths measured by experimental and theoretical methods.

Since the contact regions obtained using the experimental method were not fully filled, a comparison of contact pattern widths was carried out for the CAD and experimental methods for the same gear configuration. The contact width $a_{h}$ was measured at the mid-height of the contact patterns. The comparison is presented in Figure 28.

**Figure 29:**
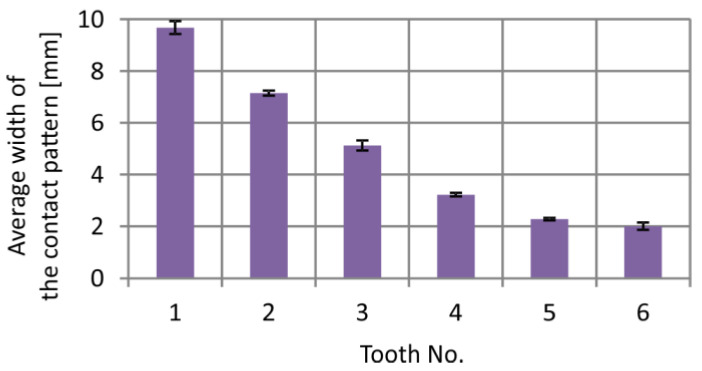
Average width of the contact pattern based on results obtained with CAD method at δ=20 μm, and with experiment—standard deviation.

The highest convergence of results was obtained at δ=20 μm. Regarding the contact pattern widths, the difference between experimental and CAD methods averages 11% (Fujifilm) and 9% (Sensor Products Inc.). Based on the results from Figure 28, a graph was developed to present the average values obtained from the CAD method at δ=20 μm and experimental measurements (using Fujifilm and Sensor Products Inc.) for each contact pattern (Figure 29). The average values were supplemented with standard deviations.

The presented methods for determining the contact pattern are particularly useful for engineers and researchers conducting studies on new types of tooth profiles. The theoretical method should be applied during the design stage of the geometry of meshing gears. However, the experimental method can be used during prototype manufacturing, the assembly of the actual gearbox, and for periodic inspection of the correct gear meshing. The obtained results allow the user to properly adjust the gearbox wheels so that the position of the contact pattern ensures safe and stable operation of the transmission.

In the analysed case, the theoretical method allowed us to verify whether the size and position of the contact pattern were correct. The results showed that the contact pattern is located near the centre of the rim width. Furthermore, it was not observed that the contact pattern shifted to the rim edge, which could lead to unfavourable edge effects—and consequently, to rim chipping and even gearbox destruction.

Subsequently, the verified models were used to manufacture a gearbox prototype. The application of the experimental method then allowed for the validation of the theoretical method, demonstrating that the shape and position of the contact pattern were consistent with the expectations from the theoretical studies.

Further research will include determining the pressure distribution in the contact zone and the shape and position of the contact pattern in a gearbox made from typical materials used in worm gear construction and studying the mentioned gear meshing parameters at different stages of rim wear.

## 4. Conclusion

In this article, the presented methods were applied to a globoid worm gear with a rectilinear axial tooth profile of the worm. The results of the investigations and model verification confirm the usefulness of the developed approaches for tooth contact analysis. Based on both theoretical and experimental observations, the following conclusions were formulated:

- The CAD-based method for determining the contact pattern can be successfully applied to evaluate the form, size, and position of the contact pattern.
- The novel method of contact area measurement using two-sheet pressure measurement film can be successfully applied to evaluate the size and position of contact patterns.
- The experimental method using chalk paint can be applied under workshop conditions, without the need for digital analysis of scanned samples, to quickly assess the correctness of gearbox assembly
- Differences in the resulting contact pattern areas depend, among other factors, on the penetration depth in the CAD method and the intensity threshold used in the digital image processing of experimental data.
- The developed image analysis algorithm enables contact pattern evaluation using a standard office scanner, eliminating the need for dedicated scanning devices and proprietary software typically offered by pressure film manufacturers.

The proposed methods provide a solid foundation for further analysis and improvements of tooth contact performance in this type of gear mechanisms.

## Figures and Tables

**Table 1:** Data of analysed gear pair.

Parameter	Worm	Worm Wheel
Normal module [mm]	$m_{n}=7.1365$	
Number of teeth [–]	$z_{1}=1$	$z_{2}=46$
Normal pleasure angle [°]	$\alpha_{n}=20^{{}^{\circ}}$	
Base diameter [mm]	$d_{b}=124.9464$	
Pitch diameter [mm]	$d_{w1}=70$	$d_{w2}=330$
Throat diameter [mm]	$d_{a1}=80$	$d_{a2}=340$
Root diameter [mm]	$d_{f1}=57.7$	$d_{f2}=317.7$
Outside diameter [mm]	$d_{e1}=104.9$	$d_{e2}=347$
Effective worm thread length [mm]	$b_{1eff}=120.1$	–
Face width [mm]	$b_{1}=135$	$b_{2}=57$
Length of flat on outside diameter	$b_{f1}=4$	$b_{f2}=2$
Face angle [°]	$\xi_{1}=35^{{}^{\circ}}$	$\xi_{2}=35^{{}^{\circ}}$
Root form radius [mm]	–	$r_{f2}=42.3$
Throat form radius [mm]	$r_{g1}=169.6$	$r_{g2}=32$
Centre distance [mm]	a=200	

**Table 2:** RDG720 material parameters [52].

Parameter	Value
Modulus of elasticity [MPa]	2000
Ultimate tensile strength [MPa]	50
Density [g/cm^3^]	1.185
Elongation at break [%]	15
Hardness Shore D	83

**Table 3:** Contact pattern areas determined in the CAD environment.

	φ_1_ [°]											
	−26	0	36	72	108	144	180	216	252	288	324	334
No.	Contact Pattern Area [mm^2^]											
1	0.0	148.2	146.4	140.3	129.4	123.9	119.2	116.7	114.0	109.3	105.4	104.3
2	104.3	101.4	98.0	94.7	91.5	88.6	85.5	82.7	79.9	77.1	74.5	73.8
3	73.8	71.8	69.1	66.6	64.0	61.6	59.2	56.9	54.8	52.4	50.0	49.3
4	49.3	47.6	45.3	43.1	41.0	39.0	37.3	35.3	33.9	32.2	31.0	30.7
5	30.7	29.7	28.4	27.6	26.5	25.9	25.2	24.7	24.4	24.2	24.2	24.1
6	24.1	24.1	24.0	24.2	24.5	24.7	25.1	25.3	26.1	24.5	0.0	0.0
**Σ**	282.1	422.7	411.2	396.4	376.8	363.7	351.5	341.6	333.2	319.7	285.0	282.1

## Data Availability

The original contributions presented in this study are included in the article. Further inquiries can be directed to the corresponding author.
